# Association between dietary inflammation index and hypertension in participants with different degrees of liver steatosis

**DOI:** 10.1080/07853890.2023.2195203

**Published:** 2023-04-10

**Authors:** Wenhao Wu, Zhuoya Zhang, Yan Qi, Hua Zhang, Yuan Zhao, Jin Li

**Affiliations:** a Department of Endocrinology and Metabolism, The Second Hospital of Shanxi Medical University, Shanxi Medical University; b Department of Biochemistry and Molecular Biology, School of Basic Medicine, Shanxi Medical University

**Keywords:** Dietary inflammatory index, non-alcoholic fatty liver disease, liver steatosis, hypertension, blood pressure, national health and nutrition examination survey

## Abstract

**Background:**

The prevalence of hypertension (HTN) is higher in patients with non-alcoholic fatty liver disease (NAFLD). Inflammation is the key link between HTN and NAFLD. Systemic inflammation can be dramatically increased by inflammatory diet intake. However, whether controlling the inflammatory diet intake in NAFLD patients could affect the occurrence of HTN still remains unknown. Our aim here is to evaluate the effect of the dietary inflammatory index (DII) on blood pressure in patients with different grades of hepatic steatosis.

**Materials and Methods:**

The data were collected from the National Health and Nutrition Examination Survey (NHANES) (2017-2018). DII was calculated based on the data of 24-h dietary recall interviews. The severity of liver steatosis was assessed by a controlled attenuation parameter. Multivariable logistic regression, multivariable linear regression and subgroup analyses were conducted to determine the association between DII and blood pressure in patients with different degrees of hepatic steatosis.

**Results:**

A total of 5449 participants were included in this analysis. In male participants with severe liver steatosis (S3), the highest DII tertile group was more likely to have higher systolic blood pressure (SBP) compared with the lowest tertile group (Tertile1: 128.31(125.31,131.31), Tertile3: 133.12(129.40,136.85), P for trend =0.03551). DII was positively correlated with SBP and the prevalence of HTN in males with hepatic steatosis grade S3 (≥ 67% steatosis) (SBP: P for trend = 0.011, HTN: P for trend = 0.039). Regarding the association of DII with SBP and HTN, the tests for interaction were significant for hepatic steatosis (SBP: interaction for *p* = 0.0015, HTN: interaction for *p* = 0.0202).

**Conclusions:**

In the present study, we demonstrated that DII was a risk factor for increased SBP and the prevalence of HTN in males with severe hepatic steatosis S3, indicating that anti-inflammatory dietary management should be considered in these individuals to reduce the risk of developing HTN.

## KEY MESSAGE

- Patients in the highest DII tertiles group had a higher SBP and higher rates of HTN compared with patients in the lowest DII tertiles group in male participants with severe hepatic steatosis S3.
- DII was positively associated with SBP and the prevalence of HTN in males with hepatic steatosis grade S3.
- The effect of DII on SBP and prevalence of HTN was significantly dependent on the severity of hepatic steatosis.

## 1. Introduction

Non-alcoholic fatty liver disease (NAFLD) is a major public health problem worldwide [1]. NAFLD is defined as a pathological condition with excessive lipid accumulation in the liver, primarily due to the impact of overnutrition in individuals without secondary causes such as significant alcohol intake and viral infections. It is a disease spectrum ranging from simple steatosis to non-alcoholic steatohepatitis (NASH) [2,3]. The prevalence of NAFLD is gradually increasing, reaching as high as 37.1% in the general adult population in western countries [4]. Notably, NAFLD is recognized as a multi-system disease affecting various extrahepatic organs, subsequently leading to high morbidity and mortality.

Hypertension (HTN), one of the increasingly serious public health problems, is a multifactorial disease caused by the interaction of genetic susceptibility and environmental impacts [5]. HTN is one of the strongest risk factors for cardiovascular disease (CVD) [6]. Several studies have shown that blood pressure is positively associated with the presence of NAFLD, and 49.5% of hypertensive patients have NAFLD [7–9]. Furthermore, epidemiological evidence demonstrates that the prevalence of HTN in patients with NAFLD is higher than that in the general population [8,10,11]. Another prospective cohort study of Framingham Heart Study also showed a similar association between NAFLD and HTN [12]. Although these studies suggest a bi-directional relationship between the two diseases, whether and how the presence of NAFLD affects the occurrence of HTN remains unclear.

NAFLD can cause a variety of systemic adverse responses, including local and systemic inflammatory responses, insulin resistance, activation of renin-angiotensin system (RAS) and sympathetic nervous system (SNS) which are important pathophysiological mechanisms of HTN [13,14]. Among them, inflammatory responses in NAFLD play a critical role in mediating the progression of HTN. NAFLD is associated with systemic inflammatory responses characterized by elevated circulating levels of tumor necrosis factor-α (TNF-α), interleukin-6 (IL-6) and CC-chemokine ligand 2 (CCL2) [15]. Furthermore, local inflammatory responses in NAFLD in blood vessels, kidneys and adipose tissues can directly accelerate the onset and progression of HTN [16–19].

Dietary inflammatory index (DII) is a literature - and population-based scoring system designed to assess the inflammatory potential of diet [20]. A positive value of DII corresponds to a pro-inflammatory diet, and a negative value of DII corresponds to an anti-inflammatory diet. The intake of pro-inflammatory diet significantly increases circulating inflammatory markers, such as TNF-α and IL-6 [21,22], total white blood cells as well as neutrophils [23]. Previous studies have shown that DII is significantly associated with the risk of various cancers [24,25], obesity [26], hyperparathyroidism [27] and osteoporosis [28]. However, whether controlling the inflammatory diet intake in NAFLD patients could affect the occurrence of HTN still remains unknown. The aim of the present study is to evaluate the effect of DII on blood pressure in patients with different grades of hepatic steatosis using data from National Health and Nutrition Examination Survey (NHANES).

## 2. Materials and methods

### 2.1. Data sources

**Figure 1.**
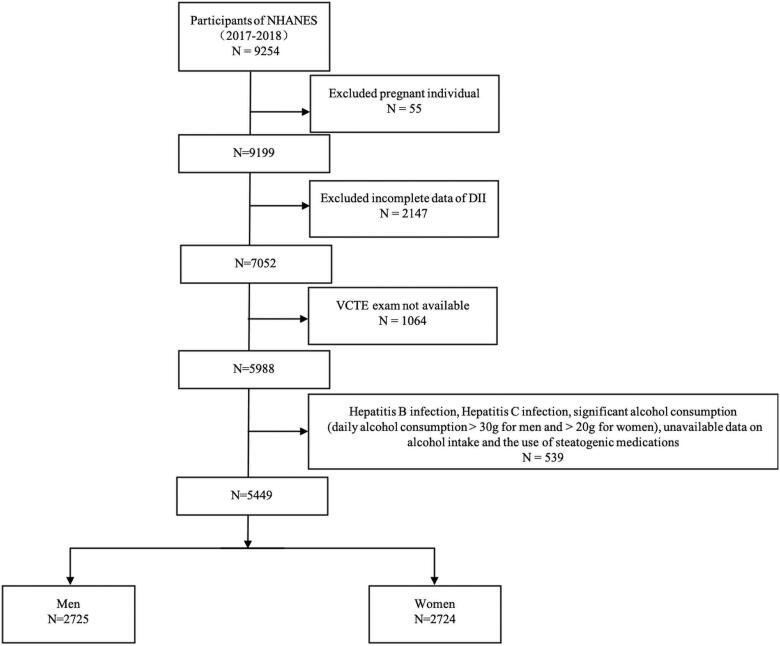
Flow chart of participants in this study.

This study was based on the data from 2-year NHANES surveys from 2017 to 2018 and all NHANES data are publicly available [29]. A total of 9254 participants were enrolled at first; after the exclusion of individuals who were pregnant (*n* = 55), missed the dietary data relating to DII (*n* = 2147), had an incomplete vibration controlled transient elastography (VCTE) exam (*n* = 1064), and had other causes for liver injury such as Hepatitis B infection, Hepatitis C infection, significant alcohol consumption (daily alcohol consumption >30 g for men and >20 g for women) [30], unavailable data on alcohol intake and the use of steatogenic medications (*N* = 539), 5449 participants were included in our final analysis (Figure 1). The National Center for Health Statistics (NCHS) Ethics Review Board granted the human subject approval for the conduction of NHANES and written informed consents were obtained from all the participants.

### 2.2. Vibration controlled transient elastography (VCTE) and classification of liver steatosis

VCTE is a well-validated non-invasive technique assessing the presence of liver steatosis through the controlled attenuation parameter (CAP) [31]. The exam was performed by NHANES technicians after a 2-day training program with an expert technician. CAP values were presented in decibels per meter (dB/m). Based on the recent study by Eddowes et al. [32], cutoff values of CAP for different degrees of hepatic steatosis were 302 dB/m, 331 dB/m, and 337 dB/m, respectively. Participants with CAP < 302 dB/m were defined as the group S0, which means hepatic steatosis <5%. Participants with CAP ≥ 302 dB/m were defined as group S1, which means hepatic steatosis ≥ 5%. Participants with CAP ≥ 331 dB/m were defined as group S2, which means hepatic steatosis ≥34%. Participants with CAP ≥ 337 dB/m were defined as the group S3, which means hepatic steatosis ≥67%.

### 2.3. Definition of blood pressure

Blood pressure (BP) was measured by trained health workers following the standardized program. The value of BP was the mean of 3 consecutive measurements collected after 10 mins of seated rest using standard mercury sphygmomanometers. HTN was defined as systolic blood pressure (SBP) > =130mm Hg and/or diastolic blood pressure (DBP) > =80mm Hg and/or a history of HTN or current use of antihypertensive drugs [33].

### 2.4. Dietary inflammatory index (DII)

DII is a new tool to assess the potential risk of an entire diet on inflammation. It is based on extensive literature including animal and epidemiological studies to investigate the inflammatory potential of diet. The inflammatory effect of diet means it could significantly increase the circulating levels of interleukin-1β (IL-1β), IL-6, TNF-α or C-reactive protein (CRP), or decrease the circulating levels of interleukin-4 (IL-4) or interleukin-10 (IL-10) [20]. Calculation of the DII scores for each individual was based on the data of 24-h dietary recall interviews and this score could evaluate the inflammatory potential of diets. Higher positive DII scores indicated pro-inflammatory diets and lower negative DII scores indicated anti-inflammatory diets [20]. Food parameters that were used to calculate DII scores in this analysis included anti-inflammatory food parameters (alcohol, vitamin B-6, b-carotene, caffeine, Eugenol, fibers, folic acid, garlic, ginger, magnesium, monounsaturated fatty acid, niacin, n-3 fatty acid, n-6 fatty acid, onion, polyunsaturated fatty acid, tiboflavin, taffron, selenium, thiamin, turmeric, vitamin A, vitamin D, vitamin C, vitamin E, zinc, green/black tea, flavan-3-ol, flavones, flavonols, flavonones, anthocyanidins, isoflavones, pepper, thyme/oregano and rosemary) and pro-inflammatory food parameters (cholesterol, carbohydrates, energy, fats, iron, vitamin B-12, protein, and saturated fat).

### 2.5. Other study variables

Other variables including age, race, body mass index (BMI), smoking status, physical activities, total energy intake and diabetic status were collected. Race were categorized as the white race, the black race, the Mexica race and other race. BMI was calculated as weight in kilograms divided by height in meters squared. Physical activity was calculated based on a detailed physical activity survey described previously [34]. Total energy intake was calculated from three-day dietary-recall food composition tables. Diabetes mellitus (DM) was defined as if any of the following conditions were met: 1) DM was diagnosed by doctor, 2) glycosylated hemoglobin A1c (HbA1c) (%) > 6.5, 3) fasting glucose (mmol/l) > = 7.0, 4) random blood glucose (mmol/l) > = 11.1, 5) two-hour blood glucose in an oral glucose tolerance test (OGTT) (mmol/l) > = 11.1, 6) the use of anti-diabetic medications. CVD was defined as a composite of coronary artery diseases, stroke or transient ischemic attacks. Chronic kidney disease (CKD) was defined as the presence of either albuminuria or low estimated glomerular filtration rate (eGFR) (< 60 ml/min/1.73 m^2^) according to KDIGO 2021 Clinical Practice Guideline for the Management of Glomerular Diseases [35]. The presence of albuminuria was defined as urinary albumin/creatinine ratio (ACR) > 30 mg/g. Laboratory methods for measurements of HbA1c, glucose, total cholesterol (TC), high density lipoprotein (HDL), low density lipoprotein (LDL), alanine aminotransferase (ALT), aspartate aminotransferase (AST) is reported in detail elsewhere [36].

### 2.6. Statistical analysis

All analyses were conducted using R version 3.4.3 (http://www.R-project.org, The R Foundation). Appropriate weighting for each analysis was applied as suggested by the NCHS. Continuous variables were presented as numbers and weighted proportions. Categorical variables were described as means (95% confidence intervals [CIs]) and percentages (95% CIs). Either weighted Student’s *t*-test (for continuous variables) or weighted chi-square test (for categorical variables) was conducted to determine the statistical significance. To examine the association between DII tertiles and blood pressure levels in different degrees of hepatic steatosis, weighted multivariable linear regression was performed. To assess the association between DII tertiles and HTN in different degrees of hepatic steatosis, weighted multivariable logistic regression was conducted. In model 1, no covariates were adjusted. Model 2 was adjusted for age and race. Model 3 was adjusted for race, age, TC, physical activity, energy intake, BMI, smoking, ALT, AST, HbA1c, HDL, LDL, CVD and CKD. To further verify the association of DII with blood pressure levels and HTN in three models mentioned above, weighted multivariable logistic regression was conducted. An interaction analysis was applied to evaluate the heterogeneity of associations among different degrees of hepatic steatosis. To explore the association of DII with SBP and HTN in different male subgroups, stratified weighted multivariate regression analysis was performed. *p*-values < 0.05 was considered statistically significant.

## 3. Results

### 3.1. Characteristics of Participants included

The basic characteristics of included subjects according to different liver steatosis levels were listed in Table 1. A total of 5449 participants were included in this analysis, consisting of 2725 (50.1%) males and 2724 (50.9%) females. In males, compared with S0, patients with S1 and above were more likely to be older, non-Hispanic white and had higher BMI, SBP, DBP, circulating levels of ALT, AST, TC, HDL, HbA1c and lower physical activity. There were no differences in energy intake, smoking status, DII and circulating LDL levels between these groups. In addition, male individuals with S1 and above were more likely to be associated with the occurrences of HTN, DM, CVD and CKD than those with S0. Females with S1 and above were significantly older and had higher BMI, SBP, DBP, circulating levels of ALT, HDL, HbA1c compared with subjects with S0. There were no differences in the race-ethnicity distribution, energy intake, physical activity, smoking status, DII and circulating LDL, TC, AST levels. Furthermore, higher occurrences of DM, HTN and CKD were observed in female subjects with S1 and above. The prevalence of CVD was similar between these different groups in females.

**Table 1.** Characteristics of included participants according to different liver steatosis level, weighted.

Characteristic	Liver steatosis level (Males)					Liver steatosis level (Females)				
	Overall(*n* = 2725)	S0 (*n* = 1891)	S1 (*n* = 834)	S2 (*n* = 494)	S3 (*n* = 441)	Overall (*n* = 2724)	S0 (*n* = 2143)	S1 (*n* = 581)	S2 (*n* = 306)	S3(*n* = 259)
**Age(y)** ^a^	43.02(41.67,44.38)	40.21(38.83,41.60) Ref.	49.20(47.50,50.90) <0.0001	49.47(46.87,52.06) <0.0001	48.95(46.49,51.40) <0.0001	45.00(43.51,46.49)	43.49(41.84,45.13) Ref.	51.02(49.28,52.76) <0.0001	51.03(48.95,53.11) <0.0001	50.73(48.51,52.95) <0.0001
**Race (%)** ^b^		Ref.	<0.0001	0.007	0.006		Ref.	0.17	0.34	0.47
Non-hispanic white	62.65(54.84,70.47)	61.46(56.45,66.47)	65.27(58.18,72.36)	65.89(57.65,74.13)	66.63(58.26,75.01)	62.16(53.86,70.46)	62.45(57.20,67.70)	61.01(52.94,69.09)	61.80(53.49,70.11)	64.20(54.95,73.44)
Non-hispanic black	10.68(8.27,13.09)	12.38(9.30,15.45)	6.96(4.51, 9.42)	7.13(4.64, 9.63)	6.98(4.53, 9.43)	11.80(8.62,14.99)	12.27(9.18,15.36)	9.94(5.64,14.24)	9.12(5.09,13.14)	9.04(4.64,13.43)
Mexican American	10.04 (6.20,13.88)	8.63(5.53,11.73)	13.14(7.84,18.4)	12.22(6.76,17.67)	12.37 (6.84,17.90)	8.99(6.05,11.93)	8.35(5.93,10.77)	11.54(6.58,16.50)	10.52(5.87,15.18)	10.18(5.46,14.89)
Other hispanic	16.62(13.59,19.66)	17.53(14.19,20.88)	14.62(11.69,17.55)	14.77(11.82,17.72)	14.02(11.10,16.93)	17.05(13.68,20.41)	16.93(13.01,20.85)	17.51(12.23,22.78)	18.56(11.85,25.27)	16.59(9.47,23.71)
**Alt (mg/dL)^a^**	26.89(25.46,28.32)	23.59(22.14,25.04) Ref.	34.02(31.35,36.69) <0.0001	38.20(33.97,42.44) <0.0001	39.00(34.31,43.68) <0.0001	18.24(17.72,18.76)	16.71(15.95,17.46) Ref.	24.22(22.21,26.23) <0.0001	25.02(21.96,28.08) <0.001	24.89(21.40,28.39) <0.001
**Ast (mg/dL)^a^**	24.22(23.13,25.31)	23.27(22.09,24.45) Ref.	26.27(24.28,28.26) 0.015	28.08(25.28,30.89) 0.003	28.44(25.32,31.55) 0.004	19.97(19.42,20.51)	19.42(18.81,20.03) Ref.	22.08(20.55,23.61) 0.009	22.21(20.05,24.37) 0.031	21.92(19.43,24.42) 0.082
**TC (mg/dL)^a^**	181.76 (178.01,185.50)	178.53 (174.62,182.44) Ref.	188.71 (184.28,193.13) <0.001	187.33 (181.21,193.45) 0.002	187.98 (182.75,193.21) 0.001	189.62 (185.79,193.46)	188.24 (184.47,192.01) Ref.	195.00 (189.31,200.69) 0.014	192.80 (185.19,200.41) 0.21	193.59 (185.95,201.23) 0.139
**HbA1c (%)** ^a^	5.64(5.59,5.70)	5.46(5.42,5.51) Ref.	6.03(5.95,6.10) <0.0001	6.14(6.02,6.26) <0.0001	6.13(6.01,6.255) <0.0001	5.61(5.57,5.64)	5.47(5.44,5.49) Ref.	6.16(6.01,6.30) <0.0001	6.381(6.129,6.634) <0.0001	6.420(6.133,6.707) <0.0001
**HDL (mg/dL)** ^a^	48.40(47.72,49.08)	50.75(50.13,51.37) Ref.	43.34(42.12,44.57) <0.0001	42.57(40.92,44.21) <0.0001	42.69(41.01,44.37) <0.0001	58.26(56.98,59.53)	60.11(58.81,61.41) Ref.	51.06(48.93,53.19) <0.0001	50.97(47.54,54.40) <0.0001	50.57(46.71,54.43) <0.001
**LDL (mg/dL)** ^a^	107.44 (103.90,110.98)	105.36 (101.15,109.57) Ref.	112.81 (106.91,118.71) 0.05	108.89 (99.62,118.17) 0.476	111.58 (103.41,119.74) 0.163	109.73 (106.12,113.35)	108.34 (104.83,111.86) Ref.	114.86 (106.70,123.03) 0.14	111.07 (100.35,121.78) 0.64	111.40 (100.24,122.57) 0.61
**CAP (dB/m)^a^**	267.41 (263.64,271.19)	232.09 (229.48,234.71) Ref.	345.03 (340.23,349.82) <0.0001	366.31 (362.88,369.74) <0.0001	370.43 (366.88,373.98) <0.0001	250.14 (247.07,253.20)	227.59 (224.22,230.96) Ref.	339.82 (336.64,343.01) <0.0001	362.05 (357.44,366.65) <0.0001	365.40 (360.17,370.62) <0.0001
**BMI (kg/m2)^a^**	28.90(28.32,29.49)	26.54(26.00,27.07) Ref.	34.09(33.15,35.04) <0.0001	35.80(34.79,36.81) <0.0001	36.15(35.05,37.26) <0.0001	29.21(28.54,29.88)	27.42(26.78,28.06) Ref.	36.29(35.14,37.45) <0.0001	37.91(36.65,39.17) <0.0001	38.31(37.08,39.53) <0.0001
**Energy intake** **(kcal/d)** ^a^	2518.25 (2470.80,2565.7)	2506.48 (2454.04,2558.92) Ref.	2544.12 (2465.54,2622.71) 0.39	2560.55 (2456.84,2664.26) 0.26	2571.38 (2431.22,2711.54) 0.31	1846.03 (1796.21,1895.85)	1844.25 (1797.69,1890.81) Ref.	1853.12 (1735.07,1971.16) 0.88	1879.49 (1715.09,2043.89) 0.67	1895.58 (1722.68,2068.48) 0.56
**Physical Activity (MET–h/wk)^a^**	7261.59 (6386.39,8136.80)	7819.27 (6840.09,8798.46) Ref.	6057.35 (5108.39,7006.32) 0.002	6325.21 (4987.84,7662.58) 0.03	6058.16 (4554.52,7561.81) 0.026	4473.20 (4116.22,4830.18)	4580.28 (4225.20,4935.36) Ref.	4054.41 (3164.85,4943.97) 0.27	3733.12 (2812.76,4653.49) 0.084	3554.43 (2634.35,4474.50) 0.046
**DM = yes (%)** ^b^	14.16(14.15,14.1)	6.92(5.99,7.84) Ref.	30.13(27.82,32.4) <0.0001	33.36(30.99,35.72) <0.0001	32.06(29.53,34.59) <0.0001	12.65(12.63,12.66)	7.83(6.71,8.95) Ref.	31.81(29.35,34.27) <0.0001	39.83(35.45,44.203) <0.0001	40.83(35.90,45.76) <0.0001
**CVD = yes (%) ^b^**	8.44(6.42,10.47)	7.91(6.27, 9.55) Ref.	13.25(9.16,17.35) <0.001	14.84(9.32,20.36) <0.001	15.62(9.97,21.26) <0.001	6.00(4.53, 7.47)	6.32(4.82, 7.82) Ref.	8.55(5.18,11.92) 0.17	7.53(3.02,12.03) 0.57	7.72(2.84,12.61) 0.53
**Smoke = yes (%)** ^b^	16.91(1.90, 16.92)	20.15(18.24,22.06) Ref.	16.09(15.03,17.16) 0.06	17.28(16.15,18.41) 0.12	18.43(17.06,19.79) 0.36	13.61(13.59,13.62)	14.61(13.07,16.15) Ref.	16.23(14.71,17.76) 0.38	15.95(13.86,18.04) 0.58	14.61(12.51,16.71) 0.999
**SBP (mmHg)** ^a^	122.81 (121.74,123.89)	119.64 (118.58,120.69) Ref.	129.72 (127.68,131.77) <0.0001	131.35 (128.56,134.15) <0.0001	131.90 (129.14,134.65) <0.0001	120.43 (119.13,121.74)	118.58 (117.29,119.87) Ref.	127.84 (125.94,129.73) <0.0001	130.17 (127.38,132.96) <0.0001	130.09 (127.02,133.16) <0.0001
**DBP (mmHg)** ^a^	73.33(71.96,74.71)	71.70(69.87,73.54) Ref.	76.88(75.53,78.24) <0.001	78.14(76.58,79.70) <0.0001	78.52(77.05,79.98) <0.0001	70.28(69.38,71.18)	69.67(68.75,70.59) Ref.	72.72(71.25,74.19) <0.001	73.70(71.46,75.93) 0.003	73.79(71.47,76.10) 0.004
**HTN (%)** ^b^	50.71(46.19,55.23)	39.71(35.51,43.91) Ref.	74.88(69.79,79.98) <0.0001	80.40(74.10,86.70) <0.0001	80.71(73.96,87.46) <0.0001	42.42(37.54,47.29)	37.97(35.00,40.94) Ref.	60.10(53.29,66.90) <0.0001	65.74(56.83,74.65) <0.0001	66.38(56.71,76.04) <0.0001
**CKD (%)** ^b^	12.28(10.40,14.16)	9.48(7.81,11.15) Ref.	20.49(16.82,24.15) <0.0001	22.09(16.53,27.65) <0.0001	20.98(14.72,27.24) <0.0001	14.27(11.83,16.71)	13.70(11.39,16.01) Ref.	19.66(15.42,23.89) 0.012	21.73(15.41,28.05) 0.011	22.18(15.02,29.34) 0.011
**DII^a^**	0.69(0.56,0.82)	0.67(0.52,0.83) Ref.	0.74(0.52,0.96) 0.64	0.81(0.54,1.08) 0.351	0.81(0.52,1.10) 0.36	1.30(1.11,1.49)	1.25(1.040,1.46) Ref.	1.51(1.30,1.71) 0.043	1.59(1.30,1.88) 0.05	1.61(1.31,1.90) 0.054

^a^Mean with 95% confidence interval (95% CI).

^b^Percentage with 95% confidence interval (95% CI).

MET: metabolic equivalents.

SBP and DBP levels were increased along with the increases of liver steatosis severity in both males and females (Table 2). In males, the mean blood pressure from S0 to S3 was 119.64/71.70 mmHg, 129.72/76.88 mmHg, 131.35/78.14 mmHg, 131.90/78.52 mmHg, respectively. In females, the mean blood pressure was 119.95/69.67 mmHg, 127.84/72.72 mmHg, 130.17/73.70 mmHg, 130.09/73.78 mmHg from S0 to S3, respectively. Participants with higher degree of liver steatosis tend to have higher prevalence of HTN in both males and females (Table 2). From S0 to S3, the rates of HTN were 39.71%, 74.88%, 80.4%, 80.71% respectively in males, and 37.97%, 60.1%, 65.74%, 66.38% in females.

**Table 2.** BP and the prevalence of HTN based on different hepatic steatosis level population settings, weighted.

	Overall	DII Tertile 1	DII Tertile 2	DII Tertile 3	P for trend^c^
**Males**	*n* = 2725	*n* = 909	*n* = 908	*n* = 908	
**SBP** **(mmHg)^a^**					
S0	119.64(118.58,120.69)	121.00(119.72,122.29)	118.93(117.15,120.71)	118.22(115.99,120.45)	0.02621
S1	129.72(127.68,131.77)	127.54(124.95,130.13)	133.45(129.59,137.31)	128.77(126.47,131.06)	0.4576
S2	131.35(128.56,134.15)	127.92(124.79,131.04)	135.36(131.80,138.92)	131.18(127.49,134.87)	0.1246
S3	131.90(129.14,134.65)	128.31(125.31,131.31)	135.10(131.14,139.05)	133.12(129.40,136.85)	0.03551
**DBP** **(mmHg)^a^**					
S0	71.70(69.87,73.54)	72.47(70.42,74.52)	71.41(69.10,73.71)	70.78(68.92,72.65)	0.02084
S1	76.88(75.53,78.23)	76.86(75.07,78.65)	77.64(74.79,80.48)	76.00(74.11,77.90)	0.5829
S2	78.14(76.58,79.70)	77.77(75.20,80.34)	78.79(76.60,80.98)	77.83(76.47,79.20)	0.9654
S3	78.52(77.05,79.98)	78.10(75.52,80.68)	79.07(76.87,81.28)	78.41(77.12,79.70)	0.8298
**HTN (%)^b^**					
S0	39.71(34.31,45.11)	43.63(37.48,49.77)	37.32(30.69,43.94)	36.13(31.43,40.83)	0.05666
S1	74.88(65.21,84.56)	72.51(62.84,82.17)	75.01(65.81,84.21)	78.66(73.04,84.27)	0.2513
S2	80.40(62.90,97.90)	75.51(63.96,87.07)	80.96(73.29,88.63)	87.16(81.51,92.81)	0.01653
S3	80.71(61.55,99.87)	74.17(61.73,86.61)	83.35(75.52,91.17)	87.64(81.74,93.54)	0.0132
**Females**	*n* = 2724	*n* = 908	*n* = 908	*n* = 908	
**SBP** **(mmHg)^a^**					
S0	119.95(118.64,121.27)	117.86(115.56,120.16)	119.32(117.61,121.03)	118.70(115.87,121.53)	0.6953
S1	127.84(125.94,129.73)	126.80(123.05,130.55)	128.17(124.92,131.42)	128.65(124.63,132.67)	0.4737
S2	130.17(127.38,132.96)	130.01(123.81,136.20)	127.46(124.50,130.42)	133.43(129.88,136.99)	0.3485
S3	130.09(127.02,133.16)	129.03(122.41,135.64)	128.34(125.39,131.29)	133.12(129.14,137.11)	0.3129
**DBP** **(mmHg)^a^**					
S0	69.67(68.75,70.59)	68.94(67.73,70.15)	70.61(69.35,71.88)	69.55(67.56,71.55)	0.5528
S1	72.72(71.25,74.19)	72.43(70.15,74.71)	72.92(71.14,74.70)	72.82(69.43,76.22)	0.8515
S2	73.70(71.46,75.93)	72.70(68.92,76.49)	73.22(71.40,75.04)	75.29(71.74,78.83)	0.297
S3	73.78(71.47,76.10)	72.78(68.79,76.77)	73.60(71.88,75.32)	75.02(71.55,78.49)	0.3676
**HTN (%)^b^**					
S0	37.97(32.88,43.07)	39.29(32.79,45.78)	37.76(33.39,42.12)	36.38(28.86,43.90)	0.5876
S1	60.10(50.07,70.12)	59.92(49.31,70.53)	61.66(52.63,70.69)	58.57(44.82,72.32)	0.8623
S2	65.74(52.90,78.59)	61.17(49.51,72.82)	66.87(55.97,77.78)	69.23(52.56,85.91)	0.4105
S3	66.38(53.96,78.80)	59.48(47.92,71.04)	69.35(58.59,80.11)	70.18(51.86,88.50)	0.3299

^a^Mean with 95% confidence interval (95% CI).

^b^Percentage with 95% confidence interval (95% CI).

^c^P for trend:the highest tertile group vs the lowest tertile group.

For males, in participants with severe liver steatosis (S3), the highest DII tertile group was more likely to have higher SBP compared with the lowest tertile group (Tertile1: 128.31(125.31,131.31), Tertile3: 133.12(129.40,136.85), p for trend =0.03551), while SBP was not significantly different across DII tertiles in S1-S2 group. There were no significant differences in DBP across DII tertiles in S1–S3 group. For the prevalence of HTN, in S2 and S3 group, the highest DII tertile group were more likely to have a higher rate of HTN compared with the lowest DII tertile group (S2:87.16% vs 75.51%, p for trend = 0.01653) (S3:87.64% vs 74.17%, p for trend = 0.0132). While there was no significant difference in the prevalence of HTN between highest and lowest DII tertile in S0–S1 group. For females, neither BP nor HTN was significantly different across DII tertiles regardless of the grade of liver steatosis (Table 2).

### 3.2. Higher DII was associated with higher SBP level and higher prevalence of HTN in S3 males

Weighted multivariable regression analysis was conducted to evaluate the relationship between DII tertiles and blood pressure levels in populations with different degrees of hepatic steatosis (Table 3). DII was positively associated with SBP in S3 males (Model 1, β = 0.068, 95% CI = 0.028∼0.107, *p* = 0.003). The same phenomenon was also observed after adjusting for age and race (Model 2, β = 0.048, 95% CI= 0.003 ∼ 0.093, *p* = 0.039) and for all possible influencing factors, including race, age, TC, physical activity, energy intake, BMI, smoking, ALT, AST, HbA1c, HDL, LDL, CVD and CKD (Model 3, β = 0.118, 95% CI= 0.046 ∼ 0.189, *p* = 0.003). In addition, DII was positively associated with a risk of HTN in the S3 male group. (Model 1, OR = 2.469, 95% CI= 1.105 ∼ 5.52, *p* = 0.030; Model 2, OR =2.797, 95% CI= 1.119 ∼ 6.989, *p* = 0.030; Model 3, OR =6.870, 95% CI= 1.623 ∼ 29.087, *p* = 0.012). There was no association of DII tertiles with SBP or the prevalence of HTN in females with different grades of hepatic steatosis (Table 3).

**Table 3.** The effect of DII tertiles on BP and HTN in patients with different hepatic steatosis degrees, weighted.

	β/OR^a^ (95% CI^b^), *p* value ^c^ (Male) (*n* = 2725)			β/OR^a^ (95% CI^b^), *p* value ^c^ (Female) (*n* = 2724)			
	Model 1^d^	Model 2^e^	Model 3^f^	Model 1^d^	Model 2^e^		Model 3^f^
**SBP**							
S0	−0.041(−0.068, −0.013) 0.007	−0.022(−0.048,0.005) 0.096	−0.005(−0.078, 0.068) 0.886	0.012(−0.038,0.062) 0.608	0.028(-0.009,0.065) 0.125	0.007(−0.038,0.052) 0.735	
S1	0.016(−0.028,0.059) 0.458	0.019(−0.022,0.060) 0.330	−0.026(−0.089,0.038) 0.403	0.022(−0.039,0.082) 0.452	0.034(−0.035,0.102) 0.296	0.048(−0.062,0.158) 0.368	
S2	0.037(−0.017,0.091) 0.162	0.035(−0.013,0.082) 0.132	0.08(0.001,0.160 0.047	0.036(−0.054,0.127) 0.404	0.046(−0.049,0.141) 0.301	0.09(−0.024,0.203) 0.110	
S3	0.068(0.028,0.107) 0.003	0.048(0.003,0.093) 0.039	0.118(0.046,0.189) 0.003	0.048(−0.053,0.148) 0.326	0.056(−0.049,0.161) 0.257	0.104(−0.011,0.220) 0.073	
**DBP**							
S0	−0.037(−0.072, −0.001) 0.042	−0.021(-0.057,0.014) 0.210	−0.034(−0.168,0.100) 0.598	0.014(−0.029,0.056) 0.496	0.019(−0.022,0.059) 0.329	0.015(−0.053,0.083) 0.644	
S1	−0.026(−0.085,0.033) 0.352	−0.03(−0.093,0.034) 0.316	0.006(−0.122,0.135) 0.920	0.004(−0.084,0.093) 0.918	0(−0.089,0.090) 0.992	0.094(−0.030, 0.217) 0.126	
S2	−0.013(-0.071,0.045) 0.644	−0.018(−0.085,0.049) 0.554	0.063(−0.035, 0.162) 0.190	0.055(−0.054,0.164) 0.295	0.047(−0.065,0.160) 0.367	0.063(−0.055, 0.181) 0.266	
S3	−0.016(-0.075,0.042) 0.552	−0.021(−0.086,0.043) 0.477	0.066(−0.031, 0.164) 0.168	0.053(−0.060,0.165) 0.331	0.045(−0.075,0.166 0.417	0.073(−0.044, 0.190) 0.198	
**HTN**							
S0	0.731(0.512, 1.043) 0.0794	0.894(0.527,1.516) 0.6421	0.685(0.239, 1.958) 0.454	0.884(0.54,1.447) 0.597	1.204(0.635,2.282) 0.528	1.051(0.465,2.374) 0.898	
S1	1.398(0.743,2.629) 0.27304	1.646(0.796,3.401) 0.1549	0.834(0.214, 3.256) 0.781	0.946(0.472,1.895) 0.865	1.036(0.354,3.029) 0.942	0.709(0.210, 2.389) 0.555	
S2	2.202(1.069,4.534) 0.035	2.64(1.212,5.751) 0.020	2.735(0.821, 9.107) 0.095	1.428(0.559,3.648) 0.426	1.589(0.443,5.696) 0.433	1.888(0.232,15.394) 0.528	
S3	2.469(1.105,5.52) 0.030	2.795(1.136,6.873) 0.030	6.870(1.623, 29.087) **0.012**	1.603(0.561,4.579) 0.349	1.739(0.405,7.465) 0.413	1.385(0.136,14.065) 0.768	

^a^β: effect sizes; OR: odds ratio.

^b^95% CI: 95% confidence interval.

^c^*p* value: comparison between Tertile 1 and Tertile 3.

^d^Model 1: no covariates were adjusted.

^e^Model 2: adjusted for age, and race.

^f^Model 3: adjusted for race, age, TC, physical activity, energy intake, BMI, smoking, ALT, AST, HbA1c, HDL, LDL, CVD and CKD.

**Figure 2.**
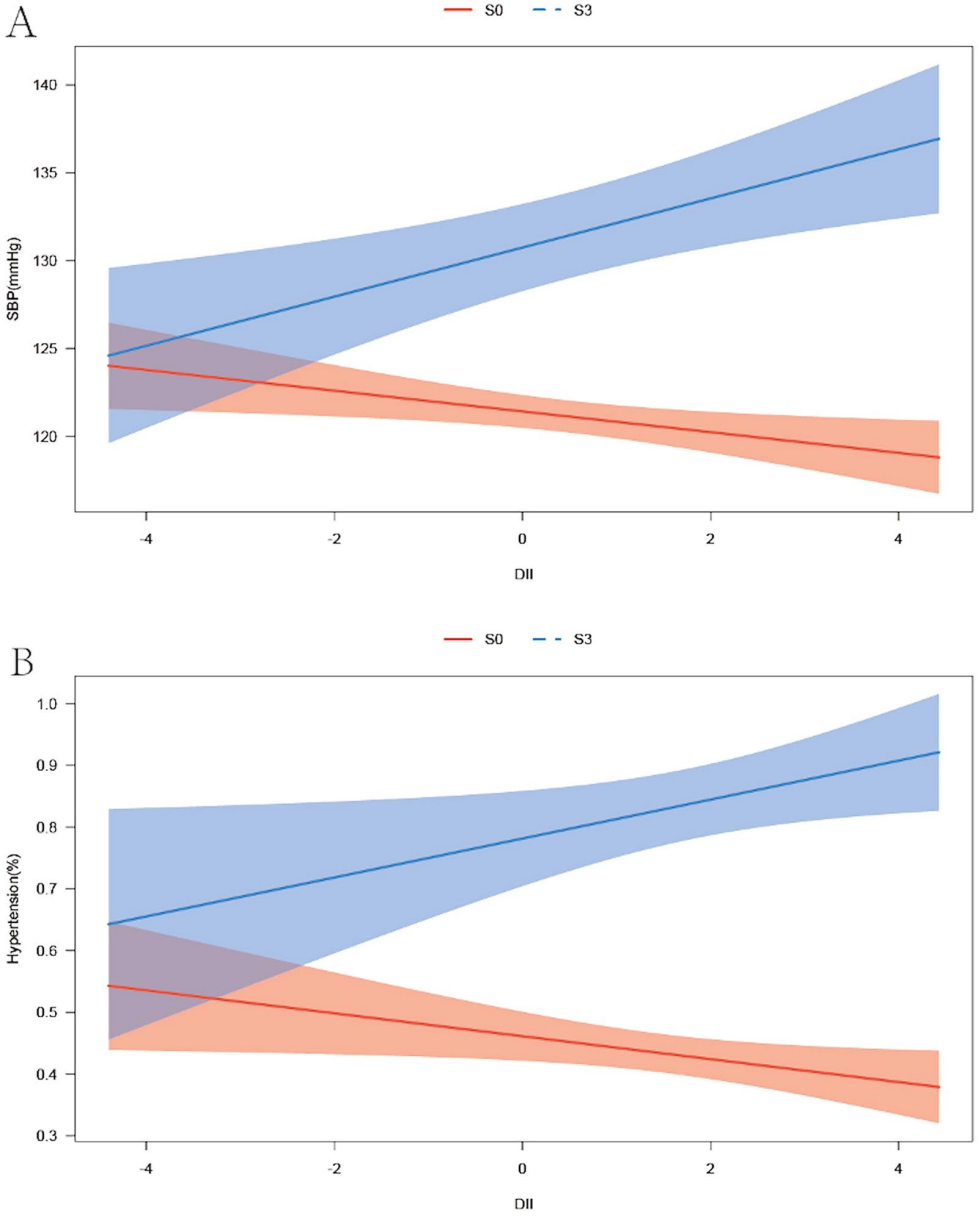
Association of DII with SBP and prevalence of HTN in S0 and S3 subgroup in males. A: Association of DII with SBP in S0 and S3 subgroup in males; B: Association of DII with prevalence of HTN in S0 and S3 subgroup in males.

The correlation between DII and SBP in S3 males also persisted when DII was considered as a continuous variable (Table 4). DII was positively associated with higher SBP (Model 1, β = 0.014, 95%CI=0.004 ∼ 0.024, *p* = 0.011; Model 2, β = 0.013, 95%CI= 0.003 ∼ 0.023, *p* = 0.016; Model 3, β = 0.025, 95%CI = 0.008 ∼ 0.043, *p* = 0.007). Meanwhile, the increase of DII raised the prevalence of HTN in S3 males (Model 1, OR = 1.199, 95% CI = 1.010 ∼ 1.423, *p* = 0.039; Model2, OR= 1.211, 95%CI= 1.014 ∼ 1.445, *p* = 0.037; Model 3, OR= 1.487, 95%CI = 1.030 ∼ 2.146, *p* = 0.036). Regarding the correlation of DII with SBP and HTN, the tests for interaction were significant for hepatic steatosis (SBP: interaction for *p* = 0.0015, HTN: interaction for *p* = 0.0202), suggesting that the effects of DII on SBP and prevalence of HTN were significantly dependent on the degree of hepatic steatosis in males (Table 4, Figure 2). There were no correlation between DII and SBP or HTN in females with different hepatic steatosis (Table 4).

**Table 4.** Association of DII with BP and HTN in patients with different hepatic steatosis degrees, weighted.

	β/OR (95% CI), *p* value			
	Model 1^a^	Model 2^b^	Model 3^c^	Interaction P (DII:S3)
**Male(*n* = 2725)**				
SBP	0.014(0.004,0.024) 0.011	0.013(0.003,0.023) 0.016	0.025(0.008,0.043) 0.007	0.0015
DBP	−0.005(−0.018,0.007) 0.371	−0.007(−0.021,0.008) 0.325	0.004(−0.018, 0.026) 0.706	0.5931
Hypertension(%)	1.199(1.010,1.423) 0.039	1.211(1.014,1.445) 0.037	1.487(1.030, 2.146) 0.036	0.0202
**Female(*n* = 2724)**				
SBP	0.009(−.009,0.027) 0.283	0.013(−0.012,0.037) 0.271	0.015(−0.011,0.041) 0.237	0.5701
DBP	0.007(−0.020,0.035) 0.587	0.005(−0.025,0.034) 0.731	0.021(−0.019, 0.060) 0.279	0.9929
Hypertension(%)	1.149(0.884,1.493) 0.275	1.183(0.821,1.706) 0.330	1.983(0.652, 6.033) 0.205	0.2090

^a^Model 1: no covariates were adjusted.

^b^Model 2: adjusted for age, and race.

^c^Model 3: adjusted for race, age, TC, physical activity, energy intake, BMI, smoking, ALT, AST, HbA1c, HDL, LDL, CVD and CKD.

### 3.3. Subgroup analysis in males

The subgroup analysis in males stratified by age, race, smoking status, energy intake was conducted to further explore the association of DII tertiles with BP and HTN (Table S1). We found the effect of DII on SBP was significantly dependent on age (P for interaction = 0.0068). In males who were younger than 50 years old, DII tertiles were negatively associated with SBP; only in males whose age were not less than 50 years, DII tertiles were positively associated with SBP.

## 4. Discussion

In the present study, we demonstrated that patients in the highest DII tertiles group had a higher SBP and higher rates of HTN compared with patients in the lowest DII tertiles group in S3 males with severe hepatic steatosis. DII was a risk factor for increased SBP and the prevalence of HTN in males with S3. The association of DII with SBP and HTN was still stable after adjustment for covariates. Furthermore, the effect of DII on SBP and the prevalence of HTN was significantly dependent on the severity of hepatic steatosis.

There exists a correlation between hepatic steatosis and HTN [12,13]. The increase in BP could predict the occurrence and development of NAFLD [37,38]. Moreover, in a Finnish study, individuals with hepatic steatosis had significantly higher SBP or DBP in mean 24-hour, daytime and nighttime than those without liver steatosis [39]. Another study also showed that hepatic fat fractions were positively correlated with BP and the prevalence of HTN in the general population. Similarly, our study found that SBP, DBP and the prevalence of HTN increased significantly with the increase in the severity of liver steatosis in both males and females (Table 1).

These findings suggest that NAFLD may be a risk factor for the development of HTN [40]. However, in the future, prospective studies should be performed to explore the existence of a causal relationship between these two diseases.

In the presence of hepatic steatosis, the accumulation of various immune cells in the liver is dramatically increased, subsequently releasing cytokines into circulation and inducing systemic inflammatory responses [15,41,42]. Inflammation further induces the activation of SNS and RAS [43], which are the pathological mechanisms underlying the development of HTN [43,44]. Diet intake is also one of the sources of inflammation in the body. DII is used to evaluate the dietary inflammatory potential. Interestingly, the effects of DII on BP levels seem inconsistent so far. Based on the study conducted by Ala’a et al. participants with higher DII scores had significantly lower SBP levels in European adults [42]. However, DBP levels did not vary significantly across DII tertiles in these individuals [45]. The study performed by Zhang X et al. demonstrated that the level of DII was significantly positively correlated with SBP and DBP [46]. In the present study, we demonstrated that patients in the highest DII tertiles group had lower SBP and DBP in the male S0 group. Inversely, patients in the highest DII tertiles group in S3 males had a higher SBP and higher rates of HTN. DII was also positively correlated with SBP and HTN in S3 males, and there was a significant interaction between the degree of liver steatosis and DII in this correlation, indicating the effects of DII on BP may be associated with the severity of hepatic steatosis. Therefore, it is necessary to further explore an accurate dietary scheme for male patients with severe liver steatosis to reduce the risk of developing HTN and its related cardiovascular complications.

However, the association of DII with SBP and HTN mentioned above did not observed in females, suggesting there might be other determinants in females. Previous studies have pointed out that women tend to be less susceptible to environmental stress (such as oxidative stress) than men [47]. One of the key factors mediating such difference may be sex hormones, such as estrogen. Estrogen exerts antioxidant and anti-inflammatory effects in women [48]. Therefore, eating habits may have less impact on the development of HTN in females with NAFLD.

In this study, we explored the possible effects of dietary inflammation on BP in patients with different grades of liver steatosis. The positive correlation between dietary inflammation and blood pressure was dependent on the degree of liver steatosis in males, suggesting that patients with more than 67% liver steatosis should pay more attention on inflammatory diet intake. We performed this analysis based on nationwide survey data and adopted sample weights to make the analysis more comprehensive. We also used the 2017 BP guidelines from the American College of Cardiology (ACC)/American Heart Association (AHA) in the present study which reduce the definition of hypertension from a cutoff of 140/90 mmHg [49] to 130/80 mmHg [33]. Such a focus on lower blood pressure targets may benefit patients for early interventions [50]. However, there are some limitations to our study. First, the information on DII was based on the data collected from dietary recalls, in which a recall bias was inevitable. Second, data from 24-h dietary recall interviews were used to calculate DII scores in this analysis, which does not represent seasonal variability in diet patterns. Thirdly, the use of DII evaluating the potential of dietary inflammation is not comprehensive, as not all variables in the dietary are assessed. Fourthly, it is well established that biopsy is the golden standard for diagnosing NAFLD. The degree of hepatic steatosis in this analysis was evaluated by VCTE, which may not be as accurate as biopsy-based evaluation.

## 5. Conclusions

In the present study, a significant association of DII with SBP and HTN was observed in males with hepatic steatosis ≥ 67%, indicating that anti-inflammatory dietary management should be considered in these individuals to reduce the occurrence of HTN. Future studies should be conducted to validate the potential clinical application.

## Supplementary Material

Supplemental Material

## Data Availability

Publicly available datasets were analyzed in this study. This data can be found here: www.cdc.gov/nchs/nhanes/.
