# Supplementary material for: Association between dietary inflammation index and hypertension in participants with different degrees of liver steatosis
Source: Ann Med. 2023 Apr 10;55(1):2195203. doi: 10.1080/07853890.2023.2195203 (PMC10088928; doi:10.1080/07853890.2023.2195203)
Supplement: Supplemental Material [file IANN_A_2195203_SM5245.docx]

Table S1: Subgroup analysis in males, weighted.

|  | **SBP (β^a^ (95% CI^b^))** | | | | |  | **DBP (β^a^ (95% CI^b^))** | | | | | |  | **HTN(OR^a^ (95% CI^b^))** | | | | |
| --- | --- | --- | --- | --- | --- | --- | --- | --- | --- | --- | --- | --- | --- | --- | --- | --- | --- | --- |
| **DII** | **Tertile 1** | **Tertile 2** | **Tertile 3** | **P for trend^c^** | **p interaction** |  | **Tertile 1** | **Tertile 2** | **Tertile 3** | **P for trend^c^** | | **p interaction** |  | **Tertile 1** | **Tertile 2** | **Tertile 3** | **P for trend^c^** | **p interaction** |
| **Age (years)** |  |  |  |  |  |  |  |  |  |  | |  |  |  |  |  |  |  |
| <50 | Ref. | -0.012(-0.05~0.028) | -0.047 (-0.067~0.974) | <0.001 | Ref. |  | Ref. | 0.008(-0.050,0.065) | -0.039(-0.078,0.001) | 0.053 | | Ref. |  | Ref. | 1.228(0.727,2.074) | 0.740(0.479,1.144) | 0.159 | Ref. |
| >=50 | Ref. | 0.037(-0.002,0.076) | 0.0347(0.0001,0.069) | 0.050 | 0.0068 |  | Ref. | 0.007(-0.041,0.055) | -0.011(-0.074,0.051) | 0.699 | | 0.5001 |  | Ref. | 1.278(0.787,2.073) | 1.804(0.880,3.698) | 0.099 | 0.125 |
| **Race** |  |  |  |  |  |  |  |  |  |  | |  |  |  |  |  |  |  |
| Non-hispanic white | Ref. | 0.014(-0.017,0.045) | -0.031(-0.067,0.005) | 0.085 | Ref. |  | Ref. | 0.009(-0.047,0.065) | -0.032(-0.079,0.015) | | 0.170 | Ref. |  | Ref. | 0.693(0.48, 1.001) | 0.623(0.432, 0.9) | 0.0156 | Ref. |
| Non-hispanic black | Ref. | 0.014(-0.030,0.059 | -0.002(-0.081,0.076) | 0.947 | 0.594 |  | Ref. | 0.047(-0.033,0.126) | 0.036(-0.059,0.131) | | 0.427 | 0.257 |  | Ref. | 0.889(0.517~1.53) | 0.866(0.562~1.336) | 0.487 | 0.3634 |
| Mexican American | Ref. | 0.004(-0.029,0.037) | -4e-04(-0.047,0.046) | 0.9844 | 0.176 |  | Ref. | -0.058(-0.122,0.006) | -0.064(-0.149,0.022) | | 0.125 | 0.522 |  | Ref. | 1.063(0.614~1.841) | 0.702(0.354~1.392) | 0.2675 | 0.5263 |
| Other hispanic | Ref. | 0.011(-0.058,0.080) | -0.034(-0.087,0.020) | 0.195 | 0.935 |  | Ref. | 0.013(-0.050,0.077) | -0.06(-0.131,0.011) | | 0.093 | 0.624 |  | Ref. | 0.547(0.332~0.902) | 0.488(0.287~0.831) | 0.0121 | 0.5268 |
| **Smoke** |  |  |  |  |  |  |  |  |  | |  |  |  |  |  |  |  |  |
| No | Ref. | 0.02(-0.005,0.046) | 0.005(-0.025,0.035) | 0.737 | Ref. |  | Ref. | 0.007(-0.029,0.044) | -0.012(-0.048,0.025) | | 0.501 | Ref. |  | Ref. | 0.984(0.719~1.346) | 1.124(0.703~1.797) | 0.598 | Ref. |
| yes | Ref. | 0.034(-0.007,0.075) | -0.039(-0.121,0.043) | 0.325 | 0.296 |  | Ref. | 0.03(-0.029,0.088) | 0.004(-0.104,0.111) | | 0.942 | 0.756 |  | Ref. | 0.814(0.439~1.507) | 0.7(0.394~1.244) | 0.203 | 0.266 |
| **Energy intake (kcal/d)** |  |  |  |  |  |  |  |  |  |  | |  |  |  |  |  |  |  |
| <2400 | Ref. | 0.03(-0.015,0.074) | -0.006(-0.047,0.035) | 0.755 | Ref. |  | Ref. | 0.014(-0.038,0.066) | -0.016(-0.064,0.032) | 0.474 | | Ref. |  | Ref. | 0.794(0.535~1.177) | 0.673(0.448~1.01) | 0.0549 | Ref. |
| >=2400 | Ref. | 0.005(-0.026,0.037) | -0.042(-0.091,0.007) | 0.088 | 0.276 |  | Ref. | 0.01(-0.037,0.058) | -0.014(-0.084,0.056) | 0.666 | | 0.805 |  | Ref. | 0.72(0.479~1.082) | 1.051(0.568~1.946) | 0.865 | 0.2630 |

^a^ β: effect sizes; OR: odds ratio.

^b^ 95% CI: 95% confidence interval.

^c^ P value: comparison between Tertile 1 and Tertile 3.
